# Exploring the mediating effect of creativity on the relationship between family capital and academic achievement in geography

**DOI:** 10.1038/s41598-023-48833-8

**Published:** 2023-12-06

**Authors:** Zhenni An, Jiahao Ge, Yanhua Xu, Xiaoyu Liang, Jianzhen Zhang, Mohamed Oubibi

**Affiliations:** 1 College of Teacher Education, College of Education and Human Development, Zhejiang Normal University, Jinhua, 321004 China; 2 College of Geography and Environmental Sciences, Zhejiang Normal University, Jinhua, 321004 China; 3 School of Geography and Environment, Jiangxi Normal University, Nanchang, 330022 China; 4 Smart Learning Institute of Beijing Normal University, Beijing, 100082 China

**Keywords:** Psychology, Human behaviour, Public health

## Abstract

This study examined the relationship between family capital (FC) and academic achievement in geography along with the mediating role of creativity. The main objective was to determine if FC is a positive predictor of creativity and geographic achievement, and whether creativity completely or partially mediates the relationship between FC and geographic achievement. 1268 high school students participated in this study using the Family Capital Questionnaire (FCQ), the Innovative Behavior Scale (IBS), and students’ class geography scores. SPSS 26 and Amos software were used to analyze the descriptive statistics and the correlation between the main variables. The mediating role of creativity was tested using PROCESS version 4. The correlation analyses showed that FC positively affected academic achievement in geography (β = 0.382, SE = 0.019). Creativity also demonstrated a positive effect on geography academic achievement (β = 0.376, SE = 0.022). The mediation analysis showed that creativity mediated and buffered the relationship between FC and academic achievement in geography. Thus, FC directly affected students’ academic achievement in geography and indirectly affected their creativity. This clearly demonstrates that student characteristics and the external environment should be emphasized in geography education, while placing a strong focus on cultivating individual creativity.

## Introduction

Geography courses form an important element of the 21st Century Learning Framework^1^. The importance of geography is similarly articulated in the International Charter on Geographical Education, with geography contributing to a lifelong enjoyment and understanding of our world^2^. With the world now more complex and interconnected than ever before, geography serves as an important means of gaining a comprehensive understanding of planetary and social processes and phenomena^3^. Increased academic achievement in geography contributes notably to students' geographical thinking and creative problem-solving skills^4^. For this reason, academic achievements in geography have received considerable attention in multinational educational assessments^5^, including from the National Assessment of Education Progress (NAEP), which has conducted numerous assessments of student achievement and associated trends in the study of geography^6^. The significant effect that studying geography can have on students’ development of higher-order thinking and the potential it offers for lifelong growth clearly highlights the importance of focusing on students' academic achievement in geography^7^.

In recent years, academic achievement in geography has emerged as an area of significant academic focus, and research has uncovered multiple factors that influence individual performance outcomes^8,9^. A range of internal factors such as sex, personality, cognitive ability, and interest have been shown to have a significant impact on academic achievement in geography^10^. From an external perspective, teacher professionalism is a crucial factor in determining students' difficulty of geography learning^11^. Studies have also found that a salutary external environment, bolstered by geographical practices, an urban lifestyle, encouragement, and higher expectations can contribute immensely to the nurturing of intra-individual cognition^12^. Another highly relevant research finding is that families play a considerable role in shaping individuals' internal characteristics and their ability to manage the extrinsic environment^13^.

Family capital (FC) provides various helpful resources for students' educational activities^14^. Researchers typically employ markers such as physical, human, social, and cultural capital, and socioeconomic status (SES) to gauge the impact of household capital on individual scholastic performance^15^. The correlation between FC and geography education has piqued the interest of researchers, emphasizing the integral role that FC plays in geography education^16^. Studies have also indicated that creativity is intrinsically linked to academic achievement in geography, with geography learning characterized as a highly creative process^17^. These findings suggest that FC, geographic academic achievement, and creativity are all interrelated, which provides the basis for the present study.

Although this study did not specifically explore the intricate interplay between FC, creativity, and academic achievement in geography, each of these factors point to the impact of interrelationships through the influence of both external and internal factors. First, the 4P model^18^, the Investment Theory of Creativity^19^, and the Ecology of Human Creativity^20^ all posit that creativity levels are influenced by both the immediate environment and individual personality traits. Second, researchers are keenly interested in examining how personality attributes and external factors impact student creativity^21^, with FC playing an instrumental role in student development. External environment and individual cognition have also both been identified as key predictors of academic achievement in geography^22^. Despite this, there remain notable gaps in the research that has been performed on the intrinsic linkages between these three theories and models. For example, investigations of how FC directly affects geography achievement have not been fully substantiated, while there are currently no studies that make specific arguments about the impact of creativity on FC or geography achievement. These gaps urgently need to be explored further.

This study employed a purposive sampling strategy to collect extensive data from nine public secondary schools in western China to investigate whether the hypothesized mediating relationship between FC, creativity, and academic achievement in geography holds. This research centered on two primary questions. First, does FC significantly and positively predict both creativity and academic achievement in geography? Second, to what extent does creativity function as a mediating agent in this model, partially or completely? When mediated entirely, FC augments geographic academic achievement by promoting creativity. Conversely, when used as a partial mediator, FC directly affects academic achievement in geography without affecting creativity. Therefore, the overarching objective of our research was to clarify the intricate interplay between FC, creativity, and academic achievement in geography. We are committed to building on a new research perspective. This can identify feasible ways to improve students' academic achievement in geography in terms of FC. In addition, by focusing on creativity, it provides strategic guidance for future geography instruction. Given the potential impact of sex and residential address, we considered each of these as covariate variables and controlled for them in the process. The next section discusses the definition of these three structures, the factors that influence them, and their relationships.

## Literature review

### Family capital

The concept of FC is rooted in the social capital theory, with Coleman^15^ asserting that FC comprises three distinct forms: economic, human, and social. Bourdieu introduced the notion of cultural capital, a composite sum of knowledge, skills, attitudes, and cultural backgrounds shared by individuals across all societal strata^23^. Taken together, researchers have looked more at Bourdieu’s classification to consider the effects of economic, social, and cultural capital on individuals.

The impact of FC on education has been extensively documented in previous research. Economic capital, as per Coleman’s theory^15^, is assessed through family income, which provides material resources that aid educational success. Students’ level of human capital is comparable to that of parents, who provide a cognitive environment for their children’s learning. Social capital accounts for the intimacy of parent–child bonds, which mainly influences students’ academic progress via family structure and parental involvement. Bourdieu emphasized the vital role of cultural capital, which can be embodied, objectified, or institutionalized, in the shaping of students’ academic outcomes^24^. Accordingly, it can be seen that the external or social environment generated by the family’s level of capital has a significant impact on the individual’s life circumstances^25,26^. These external influences can further influence an individual’s internal perceptions^27^, which can in turn have an affect academic achievement. This finding suggests that FC may influences academic achievement in the study of geography.

### Students' academic achievement in geography

Research has demonstrated that academic success is intricately tied to a multitude of factors, with geography being particularly influential, and having the potential to enhance students’ higher-order thinking skills^7^. By providing learners with a rich catalogue of skills and knowledge, geography engenders a geospatial perspective of the world that is vital for comprehending and navigating both local and global phenomena^28^. The GeoCapabilities project contends that its successful implementation hinges on young people's acquisition of Powerful Geographical knowledge, which is integral to advancing academic achievement in geography, and to enhancing geographic thinking and creative problem-solving^29^. These intrinsic cognitive abilities that can enable a geography-focused understanding of the world that can only be acquired through a geography curriculum^30^. In other words, the value and significance that geography provides with regards to lifelong development and academic achievement makes it an essential focus area.

Research has shown that several factors affect geography achievement. These include the level of geographic literacy and the positive attitudes of educators, both of which have been found to have a significant impact on student achievement^11^. Employing effective teaching methodologies can also improve academic performance in geography^31^. Examples include blended learning strategies, the use of conceptual maps and infographics, and engaging in field trips to facilitate deeper understanding of the subject matter and enhance skill acquisition^32^. Students' personalities^10^, spatial thinking abilities^33^ and geography learning attitudes^34^ have all been found to be significantly positively correlated with academic achievement in geography. Other notable influences on geography achievement include students' attitudes, interests, thinking ability and other internal characteristics, with other relevant extrinsic environmental factors including situations and teaching tools. While this clearly demonstrates the influence of family associations on an individual's internal cognition and external environment, to date, few studies have linked familial capital with academic achievement in geography, a shortcoming that this study aims to address. This requires us to make further research on the direct correlation between the two.

Recent studies have provided anecdotal evidence on the relevance of family level factors with regard to geography education. First, a significant correlation has been observed between elementary school students' acuity and their parents' income levels. In general, higher income levels among parents equate to greater ease in fulfilling students’ educational needs, leading to more substantive gains in geographic literacy, environmental sustainability awareness, and other related fields^35^. Second, students with highly educated parents generally score higher on geography tests^36^. Moreover, parental involvement and support have been demonstrated as being crucial to enhancing students’ academic attainment in geography^37^. Studies have also shown that families that provide their children with opportunities to travel, both domestically and internationally, can positively impact their geographic awareness and competence. Such experiences provide students with the capacity to identify place names and develop navigational skills, resulting in a more profound understanding of geography and an enhanced knowledge base^38^. FC has been shown to have a significant impact on both geography teaching and learning outcomes. This provides a strong illustration of the direct correlation between FC and geography career achievement. Based on these observations, Hypothesis 1 is formed:

#### (H1)

FC positively impacts students’ academic achievement in geography.

### Creativity

Creativity involves the unique ability to generate fresh and practical ideas or products that embody a synthesis of novelty and effectiveness^39^. Innovation research often centers on the multifaceted aspects of innovation; encompassing processes, products, individuals, and environments. Individual creative talent and the innovative environment embody the individual and social dimensions of creativity. Each has the capacity to shape an individual's creativity, impacting their internal perceptions and external surroundings, and ultimately influencing the resultant creative output^40^. Moreover, creativity has also been shown to be closely linked with thinking skills such as fluency, flexibility and originality with regard to personal cognition, imagination, induction, and deduction^41^. As creativity clearly has a significant influence on inner cognition at the individual level, educators must recognize the critical role that creativity plays in promoting innovation, and consider ways to foster and enhance this skill among students.

To attain a deeper understanding and offer a critical appraisal of creativity, it is essential to explore classical theories. Among these is Amabile's componential theory, which outlines the interconnected influence of cognitive, personality, motivational, and social factors as well as that of external circumstances on the creative process^42^. The Investment Theory of Creativity provides a similar perspective^19^, while the 4P model of creativity developed by Rhodes^18^ compactly encapsulates the multiple dimensions of creativity; outlining products, processes, persons, and places as crucial contributors to creative output. Taken together, these theories suggest that creativity arises through the intricate interplay between internal cognitive processes and the external environment.

Existing literature provides compelling evidence of the interplay between individual cognition and the external environment with regard to creativity^21^. From the standpoint of individual perception, factors such as confidence levels, self-efficacy, attitudes, and expectations have all been identified as having critical influences on creativity^43^. Bolstering emotional and intellectual intelligence can significantly enhance individual creativity, leading to greater academic achievement^44^. External environmental factors also play key roles in the development of individual creativity. Family environmental factors, such as parents’ economic and educational level, parents’ level of participation, and the school environment, as characterized by the school climate and teachers' perceptions, are all positive predictors of individual creativity development^45^.

The ecology of human creativity suggests that family environment plays a critical role in its development, primarily through the influence of individual personality traits^46^. First, when parents are more literate and economically and socially motivated, students are much more likely to be individualistic and creative^47^. A family's degree of social capital can significantly affect students’ creativity. When parents maintain harmonious relationships with their children, their attitudes, behaviors, and traits can help to foster a creative atmosphere^48^, with a supportive parenting style having been shown to positively predict creativity^49^. The existing literature does not systematically show how FC affects creativity; however, the aspects of creativity that are related to FC strongly suggest that FC is predictive of creativity. When FC provides a supportive and positive external environment for students, it positively affects students’ internal cognition and creativity, underscoring the importance of family in nurturing the development of individual creative potential. These findings demonstrate the significant predictive role of FC in enhancing creativity. This finding provides strong evidence in support of our prediction of a relationship between household capital and creativity. Thus, Hypothesis 2 is proposed:

#### (H2)

FC positively affects creativity.

While the relationship between creativity and academic achievement in geography has not received significant research attention, some studies have demonstrated that creativity can serve as a positive predictor of academic achievement in general^49^. Creativity can facilitate the acquisition, consolidation, and processing of new knowledge, including school-related knowledge^50^. A significant positive correlation has also been noted between creativity and critical and reflective thinking, with each of these variables having a significant positive predictive effect on academic performance^51^. Existing research findings also indicate that creativity can moderate a sense of place and academic performance in geography^52^. Although few studies have explicitly linked creativity to academic achievement in geography, the importance of creativity in improving academic achievement has been clearly demonstrated. This highlights the clear importance of focusing on students' creativity in geography education. These observations lead to Hypothesis 3:

#### (H3)

Creativity can positively predict academic achievement in geography.

**Figure 1:**
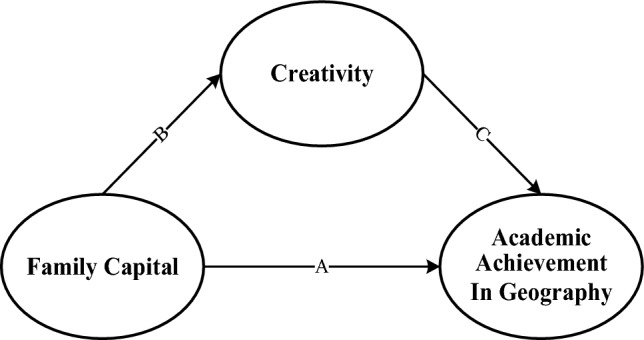
This model was formulated based on our study hypotheses and the effect of the selected variables. It describes the relationship between FC, creativity, and academic achievement in geography. This is a multiple regression model that embraces a three-path regression effect and indirect effect through the mediator creativity (**B**,**C**).

By analyzing the existing studies and taking into account the above predictions, we found that there are certain gaps in the research on the relationship between FC, creativity, and academic achievement in geography. However, there may be some connection between them. This gives us the message. We predict that FC is directly related to creativity, which in turn predicts academic achievement in geography. This leads to Hypothesis 4 (see Fig. 1):

#### (H4)

Creativity mediates the relationship between FC and academic achievement in geography.

## Methodology

### Participants and procedures

We collected data from public high schools in western China. To ensure that a balanced number of schools participated in the study, schools were selected to account for variability in urban and rural areas, city types, and school grades. A recruitment application was launched in 10 secondary schools, of whom 9 schools agreed to take part in this study; a recruitment rate of 90%. A total of 1268 participants aged 16–18 completed the survey between November 10 and 30, 2021. Study information was provided to parents, teachers, and geography teachers, and informed consent was obtained from both students and parents prior to completing the questionnaire. During recess, complete paper questionnaires were handed out to the students. Participant information was subsequently digitized and anonymized for further study. After excluding all incomplete questionnaires, the final number of valid questionnaires was 1037. The Ethics Committee College of Zhejiang Normal approved the study, which abides by the Declaration of Helsinki.

### Measurement tools

The survey used in this study had four sections: (1) demographic information, (2) FC Questionnaire, (3) Innovative Behavior Scale (IBS), and (4) Geography Academic Achievement Scoring System. The survey included the demographic items of sex, age, and residential address. To ensure that participants could remember their geography scores, we communicated with the geography teachers to ensure that the questionnaire was distributed within one week of the midterm.

#### FC questionnaire

Based on the Programme for International Student Assessment (PISA) (2018) Family Background Questionnaire, the FC Questionnaire was adjusted and modified by selecting the three dimensions of parents’ highest level of schooling, parents’ occupations, and family assets. Family cultural capital, social capital, and economic capital were surveyed, respectively. The final questionnaire consisted of six questions. First, parents' education level was rated according to their highest level of schooling. Seven points were given for a doctoral degree, six for a master’s degree, five for an undergraduate degree, four for a college degree, three for a technical school/secondary school/high school education, two for a junior high school education, and one for primary school and below. The second key factor was parents’ occupation. Five points were allocated for civil servants, organ cadres, government staff, professionals (such as teachers/journalists/stylistics/lawyers/doctors); four for business managers; three for ordinary employees (office building staff/office workers); two for commercial service workers (such as waiters/store clerks/salespersons), contractors/self-employed persons, freelancers, ordinary workers (such as manual laborers/factory workers); and one for unemployed or retired status. Third, a family's economic status was based on the number of books, equipment, learning environment, mobile phones, tablets, computers, and so on. The scoring range was 1–7, and the Cronbach’s alpha of the scale was 0.735. Based on previous research, standardized z-values for the six variables were integrated into the component analysis^53^. Principal component analysis was used to calculate household capital, with higher scores indicating higher household capital.

#### Innovative behavior scale

The Innovation Behaviour Scale follows the^54^ approach and includes three dimensions–idea generation, idea promotion, and idea realization–focusing on the processes through which people accumulate their ideas and practice innovative behavior. We used the back-translation method in this study to improve translation accuracy^55^. After discussing and agreeing on each scale’s entries individually, we translated the content into Chinese on an item-by-item basis and adjusted the entries accordingly. Each ambiguous or difficult-to-understand entry was revised in accordance with the principles of semantic equivalence. Finally, we translated the Chinese version of the scale into English and found no changes in the meaning expressed. This indicates that our translation did not negatively affect the scale’s contents. The scale consisted of 9 items (e.g., "I'll come up with an original solution to the problem (idea generation),” "I will seek the support of managers for innovative ideas (idea promotion),""I will translate innovative ideas into actionable practice (idea realization)." Responses were measured by five Likert scales ranging from 1 (never) to 5 (always), and the Cronbach alpha of the scale was 0.901.

#### Academic achievement on geography scale

**Figure 2:**
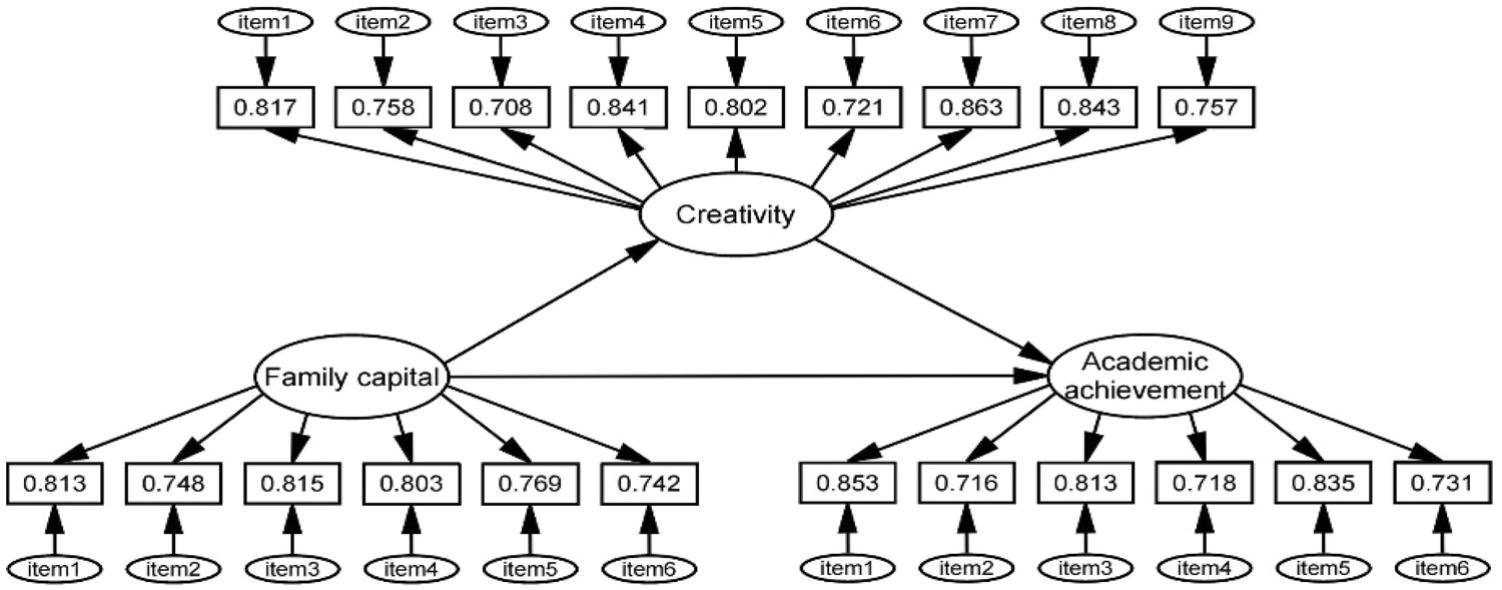
This figure displays the study model, including variables, and for each variable, it shows all the items alone with their respective factor loadings.

Given the diverse sources of our data from various schools, we developed a standardized Academic Achievement in Geography Scale to evaluate students' academic performance. This scale is rooted in the conventional grading system. Academic achievement scores in geography were categorized into six levels: grades of 40 or below, 40–59, 60–69, 70–79, 80–89, and above 90 were designated as Levels 1, 2, 3, 4, 5, 6, respectively, with corresponding point assignments of 1, 2, 3, 4, 5, 6. This approach allows for a comprehensive representation of geography students' academic accomplishments. The Cronbach's alpha of the scale was 0.776, indicating a satisfactory level of internal consistency. More details on the measurement tools and items used are shown in Fig. 2.

### Data analysis

The data relating to this study was analyzed with SPSS 26 software, AMOS, and PROCESS^56^. To ensure the validity of data analyses, Harman’s single-factor test using principal component factor analysis was performed to test the common method bias^57^. The results of the non-rotated principal component analysis showed that all three factors the eigenvalues greater > 1 and contributed 58.868% to the total variance. The first factor, at 39.458%, was below the critical threshold of 40%^58^, indicating that there was no significant common method bias. In other words, rather than varying the data collection and measurement techniques, the difference between the independent and dependent variables was mostly caused by the characteristics of the variables themselves. After evaluating the frequently utilized method deviations, a descriptive statistical analysis was conducted to compute the mean and standard deviations of the data to examine the study’s concentration and dispersion trends. Pearson’s correlation coefficients between variables were calculated to examine the degree of interrelationship and patterns of change among the independent, intermediate, and dependent variables. Finally, a mediation analysis was performed using SPSS PROCESS 4^56^ to explore the mediating role of creativity and validate the four hypotheses of the study.

## Results

### Descriptive statistics and correlation analyses

777 (74.92%) of the study participants were female and 260 (25.07%) were male. Residential addresses were urban or suburban: 621 (59.88%) interviewees lived in urban residences, and 416 (40.11%) lived in the suburbs. Table 1 shows a descriptive analysis of the respondents' FC, creativity, and academic achievement in geography. To assess the relationship between the three variables, we analyzed the Pearson correlation coefficients. First, students' FC was significantly and positively correlated with academic achievement in geography (r = 0.386, p < 0.001). Second, a significant positive correlation was found between FC and creativity (r = 0.486, p < 0.001). Finally, creativity was significantly and positively correlated with academic achievement in geography (r = 0.341, p < 0.001). The correlation results are presented in Table 2.

**Table 1:** The main variables descriptive statistics.

Variable	N	M	SD
Family capital	1037	0.0004	1.8105
Gender			
Male	260	0.1425	1.9346
Female	777	− 0.0472	1.7658
Residential address			
Urban	621	0.7774	1.7376
Suburban	416	− 1.1594	1.1920
Academic achievement in geography	1037	3.0700	1.1090
Gender			
Male	260	3.2500	1.2380
Female	777	3.0100	1.0560
Residential address			
Urban	621	3.2500	1.1320
Suburban	416	2.8000	1.0170
Creativity	1037	2.6429	0.7253
Gender			
Male	260	2.7564	0.7477
Female	777	2.6049	0.7141
Residential address			
Urban	621	2.7996	0.7508
Suburban	416	2.4089	0.6159

**Table 2:** The main variables in Pearson’s r.

Variable	1	2	3
1. Family capital	1	–	–
2. Academic achievement in geography	0.386***	1	–
3. Creativity	0.486***	0.341***	1

***p < 0.001.

### Confirmatory factor analysis

Before testing our hypotheses, we conducted a series of discriminant validity analyses of the model using a series of confirmatory factor analyses (CFAs), comparative fit index (CFI), standardized root mean square error of approximation (SRMR), Tucker-Lewis Index (TLI), goodness of fit index (GFI), expected cross-validation index (ECVI), and root mean square error of approximation (RMSEA). Additional details are provided in Table 3.

**Table 3:** Confirmatory factor analysis (CFAs).

V	CFI	TLI	SRMR	RMSEA	ECVI	GFI
FC	0.98	0.97	0.01	0.01	0.64	0.98
C	0.96	0.91	0.03	0.03	0.75	0.98
AAG	0.97	0.96	0.01	0.02	0.94	0.95

*FC* family capital, *C* creativity, *AAG* academic achievement in geography, *CFI* comparative fit index, *SRMR* standardized root mean square error of approximation, *TLI* Tucker–Lewis index, *GFI* goodness of fit index, *ECVI* expected cross validation index, *RMSEA* root mean square error of approximation.

### Mediation analysis

PROCESS V4 (Hayes, 2021) was used to conduct a simple linear regression analysis. FC was the independent variable, academic achievement in geography was the dependent variable, and creativity was the mediating variable in Model 4. To further examine the mediating role of creativity, the Bootstrap method was used to set the confidence interval (CI) at 95%. The results show that each path coefficient's 95% CI did not include zero, and the mediating effect was significant.

**Figure 3:**
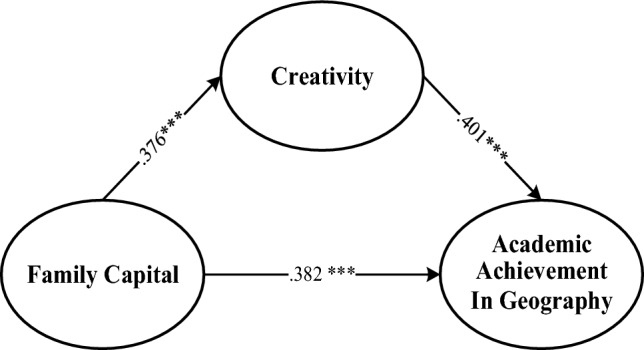
The mediation model between the main variables.

The results obtained (Fig. 3) clearly demonstrate that FC had a positive and significant impact on academic achievement in geography (β = 0.382, SE = 0.019); a positive and significant relationship with creativity (β = 0.376, SE = 0.022); and that creativity was positively related to academic achievement in geography. Each of these results are consistent with our hypotheses.

In this study, creativity positively affected the relationship between FC and academic achievement in geography. The indirect effect between FC, creativity, and academic achievement in geography was positively significant (β = 0.150, SE = 0.000, LLCI = 0.129, ULCI = 0.177). The total effect of the mediation was (β = 0.532, SE = 0.019, LLCI = 0.483, ULCI = 0.590) which is positively significant. The direct effect between FC and academic achievement in geography was also positively significant (β = 0.382, SE = 0.019, LLCI = 0.354, ULCI = 0.413). None of the CIs included 0, suggesting that creativity as a mediating variable plays a partially mediating role. Additional details are provided in Table 4.

**Table 4:** Mediation analysis for the main variables (n = 1037).

Effect	ß	SE	95.0% confidence interval	
			LLCI	ULCI
Direct effect				
FC-C	0.376	0.022	0.347	0.410
C-AAG	0.401	0.015	0.373	0.434
FC-AAG	0.382	0.019	0.354	0.413
Indirect effect				
FC-C-AAG	0.150	0.000	0.129	0.177
Total effect				
FC-C-AAG	0.532	0.019	0.483	0.590

*FC* family capital, *C* creativity, *AAG* academic achievement in geography, *LLCI* lower limit of confidence interval, *ULCI* upper limit of confidence interval.

## Discussion

This study examined the role of creativity in the relationship between family capital and academic achievement in geography. According to the findings, in our mediation model, family capital positively predicts academic achievement in geography and family capital positively affects creativity. In addition, creativity positively predicts academic achievement in geography. Most importantly, our findings reveal that creativity partially mediates the relationship between family capital and academic achievement in geography. The results of this study are consistent with the proposed hypotheses and previous findings.

First, as expected, household capital positively predicts geographic industry achievement, and the results support Hypothesis 1. Our results reveals a direct correlation between a family’s degree of cultural capital and their offspring’s level of academic achievement in geography. These results match those previously reported in parallel research in which parents’ higher educational qualifications and expectations were definitively linked to higher levels of academic achievement in geography^59^. An ancillary finding of our research is that household economic capital is an active and robust predictor of academic achievement in geography. This association has been borne out in prior literature, wherein students’ academic proficiency in geography has been unequivocally shown to be interrelated to the socio-economic status of their families^16^. In particular, families with abundant economic resources^60^ provide students with greater access to educational opportunities and resources that positively forecast their academic achievements in geography. Family social capital also plays an important role. A supportive and comfortable family environment equipped with amenities exerts a significant influence in promoting favorable academic outcomes in geography^61^. Providing students with opportunities to engage in outdoor activities, ranging from leisure walks to hikes, can invoke a sense of appreciation and fondness for scenic beauty, the natural environment, and the spirit of exploration. This kind of exposure can undoubtedly incite a lasting interest in geography^62^.

Second, as predicted, the results here were consistent with Hypothesis 2, revealing the positive effects of FC on creativity. Our research suggests that FC influences creativity development by creating an external environment that affects internal cognition. This has also been confirmed by studies that have been carried out on creative environments at home. These environments, characterized by the encouragement of novelty and diversity, inconsistency, persistence, and fantasy, as well as parental educational participation, companionship, and virtuous family relationships, create an atmosphere that is exceptionally conducive to promoting creative development among students^48^. Kaufman and Beghetto^63^ categorized creativity into four levels. Also known as the 4Cs framework—big-C, pro-C, little-c, and mini-c—they surmised that the environment manifests as a preponderant factor in determining creativity^64^. Our study further suggests that FC positively affects individuals’ intrinsic cognition by providing an environment conducive to creative development, and thus positively predicting creativity. Remarkably, these inferences align with the ecology of human creativity^46^, which states that students’ disposition towards outstanding personality traits is better when an environment is provided that is conducive to creativity. An amalgamation of environmental factors and personal characteristics, as posited in the investment theory of creativity^19^, have an undeniable impact on students’ creativity^21^.

Third, the results obtained validate Hypothesis 3, which is consistent with other studies showing that creativity positively affects academic achievement in geography. Specifically, students with higher levels of creativity consistently display higher geographic thinking skills. These competencies inevitably translate into positive academic outcomes and are significant predictors of academic achievement in Geography. Initially, the researchers explained creativity using the formula C = O ∗ TC^50^; where C represents creativity, O signifies originality, and TC refers to task constraints. Importantly, this formula indicates that creativity is a function of both originality and task constraints. Furthermore, creativity is not limited to divergent, imaginative, deductive, or inductive thinking^65^. It encompasses the ability to decipher and tackle novel problems that demand innovative solutions^66^. These findings are corroborated by Akpur^51^ who demonstrated that creative, critical, and reflective thinking had significant positive predictive effects on academic achievement. In this context, higher levels of creativity correspond to better academic performance outcomes, including with regards to geography specifically^67^. Our findings also affirm that creative thinking is a vital tenet of geography and fosters a diverse spectrum of cognitive abilities such as geospatial and scale thinking^68^. Thus, creative thinking represents a vital cognitive component that is integral to geography students’ academic success. This notion concurs with the current state of research that identifies geography as a discipline inherently aligned with creativity and imaginative thinking^69^. In other words, imagination and creative thinking can effectively influence students’ perceptions of geographical representation, positively affecting their academic achievement in geography^17^. In summary, students with higher creativity are more likely to acquire deeper geographic knowledge, better cognitive skills, and higher-level geographic thinking, which together enable greater geography achievement.

Fourth, the results are consistent with Hypothesis 4: creativity serves as a partial mediating factor linking FC and academic achievement in geography. That is, household capital directly predicts career achievements in geography. First, an environment in which parental education, expectations, financial means, involvement, and proximity are prevalent may favorably impact a student’s capacity for creativity. This is consistent with previous research highlighting the importance of such factors in fostering creativity^70^. Furthermore, creativity is a critical skill in geography learning^17^. Studies in psychology and neuroscience have linked high levels of creativity to greater bilateral hemispheric activation and brain activity^71^, which can enhance cognitive analysis and problem-solving abilities^65^. Overall, the results of this study indicate that individuals with higher levels of creativity are more active in their cognitive processes, which allows for better knowledge acquisition and problem-solving. Thus, creativity serves not only to enhance cognitive and thinking skills but also to promote the positive impact of FC on cognitive development. Creative individuals are more likely to receive positive intellectual and emotional support from their families, leading to improved academic achievement in geography. Collectively, these findings contribute to a deeper understanding of the vital role of creativity in academic success, and the ways in which it interfaces with familial and cognitive factors to shape individual performance.

## Conclusion

This investigation sought to examine the relationship between FC and academic achievement in geography among students and the mediating role of creativity in this connection. The results obtained indicate that greater levels of FC are significantly associated with improved academic performance in geography. Furthermore, creativity partially mediates the relationship between FC and academic achievement in geography. Specifically, the dimensions of FC affect creativity before affecting academic achievement in geography. These findings should prompt a deeper consideration of the importance of creativity in shaping student success in geography and the need to emphasize this attribute in school curricula and educational goals. Careful attention should also be paid to family education as the cultivation of a stimulating familial setting can significantly enhance children’s creative development. Parents should utilize positive FC to promote creativity and improve students’ performance in geography by adjusting their consumption structures and educational philosophies. Overall, these findings serve to open a meaningful avenue for exploring the factors that influence success in geography.

### Implications

This study significant expands upon and complements existing research on the study of geography, offering valuable theoretical and practical contributions, shedding light on the distinctive role FC plays in the mechanisms underlying academic success in geography. Our findings not only enrich the current understanding of the impact of FC on individual academic achievement but also clarify the factors that shape success in geography specifically. This study also establishes the pivotal role creativity plays in mediating and moderating this connection. Creativity is a highly important way of thinking with respect to geography and understanding the world in general. This study provides strong theoretical support for the development of creative thinking in geography. It aims to enrich the theoretical dimensions of higher-order geographic ways of thinking. Our study is conducive to calling for attention to the development of creativity and higher-order thinking styles in geography in geography education. Crucially, academic achievement in geography reflects the level of student learning and ability to apply geographic knowledge, making our findings highly relevant for practical applications. This study calls on geography teachers and educators to pay greater attention to the influence of FC on geography students and to continue to promote internal cognitive development. Concrete methods are identified here through which families can enhance their children’s academic performance in geography. These findings can inspire families to offer greater support to students through external educational resources and foster internal cognitive development by instilling positive educational concepts that promote creativity and boost academic achievement in geography. In summary, these findings offer essential insights into how families and educators can collaborate to optimize students’ success in geography and beyond.

### Limitations and future directions

This study possesses the following limitations. First, only high school students were selected as participants and not the entire teen population. Therefore, it remains to be determined whether this model and the results obtained can be extended to early adolescents. Second, the fact that all study participants were from western China may also have influenced the generalizability of the findings. Third, the uneven sex ratio of participants may also hinder the universalization of the results. Fourth, this was a cross-sectional study; therefore, it is difficult to draw strict causal inferences. Longitudinal data will need to be collected in future, focusing on a balanced distribution of sample cities and further clarifying the relationships and mechanisms of action between FC, creativity, and academic achievement in geography. For example, it is important to analyze which aspects of creativity act as intermediaries and which aspects of FC significantly impact academic achievement in geography.

### Ethics approval and consent to participate

The study adhered to the principles of the Declaration of Helsinki. All the participants were asked to read and sign an informed consent form. This study was approved by the Research Ethics Committee of Zhejiang Normal university (number: ZSRT2023056).

### Institutional review board statement

The study was conducted in accordance with the Decla-ration of Helsinki, and approved by the Ethics Committee of Zhejiang Normal University.

### Informed consent

Before the survey, teachers received informed consent, and the ethics committee approved all the process of the present study.

## Data Availability

The datasets used and/or analysed during the current study available from the corresponding author on reasonable request.
